# Impact of sarcomatoid differentiation and rhabdoid differentiation on prognosis for renal cell carcinoma with vena caval tumour thrombus treated surgically

**DOI:** 10.1186/s12894-020-0584-z

**Published:** 2020-02-18

**Authors:** Bin Yang, Haizhui Xia, Chuxiao Xu, Min Lu, Shudong Zhang, Guoliang Wang, Lulin Ma

**Affiliations:** 1 Department of Urology, Peking University Third Hospital, 49 North Garden Road, Haidian District, Beijing, 100191 People’s Republic of China; 2 Department of Pathology, Peking University Health Science Center, Beijing, China

**Keywords:** Sarcomatoid, Rhabdoid, Renal cell carcinoma, Vena cava, Thrombus, Prognosis

## Abstract

**Background:**

Sarcomatoid differentiation in renal cell carcinoma (RCC) with vena caval tumour thrombus has been shown to be associated with aggressive behaviours and poor prognosis; however, evidence of the impact of rhabdoid differentiation on prognosis is lacking. This study evaluated the impact of sarcomatoid differentiation and rhabdoid differentiation on oncological outcomes for RCC with vena caval tumour thrombus treated surgically.

**Methods:**

We retrospectively analysed patients treated surgically for RCC with vena caval tumour thrombus at our institute from Jan 2015 to Nov 2018. Prognostic variables were evaluated for associations with progression-free survival (PFS) and cancer-specific survival (CSS) by Kaplan–Meier survival analysis and log-rank test. Univariate and multivariate analyses were performed to determine independent prognostic variables.

**Results:**

We identified 125 patients with RCC and vena caval tumour thrombus, including 17 (13.6%) with sarcomatoid differentiation alone, 8 (6.4%) with rhabdoid differentiation alone and 3 (2.4%) with both sarcomatoid and rhabdoid differentiation. Compared to pure RCC, patients with sarcomatoid differentiation but not rhabdoid differentiation have worse PFS (*p* = 0.018 and *p* = 0.095, respectively). The univariate and multivariate analyses both showed sarcomatoid differentiation as a significant predictor of PFS. Compared to pure RCC, patients with sarcomatoid differentiation (*p* = 0.002) and rhabdoid differentiation (*p* = 0.001) both had significantly worse CSS. The univariate analysis showed sarcomatoid differentiation, rhabdoid differentiation, metastasis and blood transfusion as significant predictors of CSS (All, *p* < 0.05). In the multivariate analysis, sarcomatoid differentiation (HR 3.90, *p* = 0.008), rhabdoid differentiation (HR 3.01, *p* = 0.042), metastasis (HR 3.87, *p* = 0.004) and blood transfusion (HR 1.34, *p* = 0.041) all remained independent predictors of CSS.

**Conclusions:**

Sarcomatoid differentiation and rhabdoid differentiation are both independent predictors of poor prognosis in RCC with vena caval tumour thrombus treated surgically.

## Background

Renal cell carcinoma (RCC) is the most common kidney tumour, comprising an estimated 2.2% of all new cancer diagnoses with 403,262 new cases and 175,098 deaths in 2018 [1]. Overall 4–10% of patients with RCC present with venous tumour thrombus [2]. Sarcomatoid differentiation in RCC is characterized histologically by a dedifferentiated growth pattern of epithelial neoplasm into malignant spindle-shaped mesenchymal cells [3]. Sarcomatoid differentiation can arise in any histologic subtype of RCC; thus, it is no longer considered a distinct histologic subtype [4]. Approximately 5% of all RCCs and up to 15% of stage IV cases contain sarcomatoid differentiation [5, 6]. Previous studies suggested that sarcomatoid differentiation is associated with aggressive behaviours, poor response to targeted therapy and worse prognosis [6–8]. However, the impact of sarcomatoid differentiation on prognosis for RCC with vena caval tumour thrombus treated surgically has not been studied extensively.

Rhabdoid differentiation in RCC is characterized by “sheets and clusters of variably cohesive, large epithelioid cells with vesicular nuclei, prominent nucleoli and large paranuclear intracytoplasmic inclusions” [9]. It is present in approximately 5% of all RCCs and 27% of grade 4 RCCs [9, 10]. Rhabdoid differentiation in RCC is considered a predictor of poor prognosis, similar to sarcomatoid differentiation. Therefore, the World Health Organization International Society of Urological Pathology (WHO/ISUP) grading system formally classifies RCC with either sarcomatoid differentiation or rhabdoid differentiation as grade 4 [11]. The impact of rhabdoid differentiation in RCC on prognosis has been studied to some extent, but the available reports have inconsistent conclusions. Furthermore, there is little evidence on the prognostic role of rhabdoid differentiation in RCC with vena caval tumour thrombus treated surgically.

Therefore, this report describes the survival outcomes of a consecutive series of patients treated surgically for RCC with vena caval tumour thrombus and our evaluation of the impact of sarcomatoid differentiation and rhabdoid differentiation on survival outcomes.

## Methods

### Patients

After receiving approval from the Peking University Third Hospital Medical Science Research Ethics Committee, we retrospectively analysed the data of patients treated with nephrectomy and thrombectomy for RCC with vena caval tumour thrombus at our institute from Jan 2015 to Nov 2018. Among the 131 patients pathologically diagnosed with RCC with vena caval tumour thrombus, 6 were excluded from the study: 1 with metachronous vena caval tumour thrombus, 2 with two-stage operation for RCC with vena caval tumour thrombus and 3 with incomplete follow-up data. Thus, 125 patients were included in our study. None of the patients underwent neoadjuvant therapy before surgery. Comprehensive clinical and pathological data was collected for each patient, including age, gender, tumour size, thrombus level, blood transfusion, TNM stage, histologic subtype, Fuhrman grade, tumour necrosis, sarcomatoid differentiation, rhabdoid differentiation and adjuvant target therapy of tyrosine kinase inhibitors.

### Clinical and pathological evaluation

Tumour size was collected as the largest diameter reported in computed tomography or magnetic resonance imaging examination. The level of tumour thrombus was assigned using the Mayo classification [12]. TNM stage was determined according to the 8th edition American Joint Committee on Cancer TNM classification [13]. Histologic subtype was assigned based on the 2016 WHO classification of renal tumour [14]. The tumour grade was determined following the Fuhrman system. A commonly accepted definition of sarcomatoid differentiation and rhabdoid differentiation morphology was used [3, 9]. One urological pathologist reviewed the pathologic specimens.

### Surgical procedures

First, nephrectomy was performed following routine procedures, and lymph node dissection was performed for patients suspected to have lymph node metastasis based on enhanced CT or PET/CT results. Second, the inferior vena cava (IVC) and contralateral renal vein were isolated and blocked as follows: (a) For Mayo I tumour thrombus, the IVC tumour thrombus was squeezed back into the renal vein using the milking technique, and the IVC was partially blocked with vessel forceps. (b) For Mayo II tumour thrombus, several short hepatic veins and lumbar veins were ligated to expose the retrohepatic segment of the IVC, and the contralateral renal vein and distal and proximal IVC were blocked with rubber bands. (c) For Mayo III tumour thrombus, the liver was mobilized to expose the hepatic portal vein before blocking the IVC. (d) For Mayo IV tumour thrombus without entrance to the atrium, the milking technique and Foley catheter-assisted technique could be used to downgrade the tumour thrombus to level III. (e) For Mayo IV tumour thrombus into the atrium, thoracoabdominal midline incision and cardiopulmonary bypass were commonly necessary. Next, the junction of the renal vein and IVC was curvilinearly incised, and the tumour thrombus was pulled out once confirmed to be completely isolated. Finally, the IVC was sutured continuously after flushing the lumen with heparin saline.

### Follow-up

Follow-up was executed every 3 months for the first 2 years and semi-annually thereafter and included physical examination, laboratory tests and chest and abdomen-pelvis scans. Follow-up information was obtained through review of outpatient records and telephone calls. Progression-free survival (PFS) was calculated from the date of surgery to radiological evidence of tumour progression, death from any cause or the last follow-up. Cancer specific survival (CSS) was calculated from the date of surgery to death from RCC or the last follow-up.

### Statistical analysis

Normally distributed continuous variables were reported as means and standard deviations. Non-normally distributed continuous variables were reported as medians and interquartile ranges. The Student’s t test and Mann–Whitney U test were applied to compare continuous variables. The Chi-square test was applied to compare categorical variables. The Kaplan–Meier method with log-rank test was used for survival analysis and comparisons. Univariate and multivariate Cox proportional hazard models were performed to identify independent predictors associated with PFS and CSS. All statistical analyses were conducted with SPSS Statistics 22.0 (IBM Corp, Armonk, NY, USA). Two-tailed tests were used for all comparisons, and *p* < 0.05 was considered statistically significant.

## Results

### Baseline characteristics

A total of 125 patients treated surgically for RCC with vena caval tumour thrombus were included in our study. Thrombus levels were Mayo I, II, III, and IV in 38, 49, 25 and 13 patients, respectively. Among those patients, 17 (13.6%) had sarcomatoid differentiation alone, 8 (6.4%) had rhabdoid differentiation alone and 3 (2.4%) had both sarcomatoid and rhabdoid differentiation. The patients’ clinicopathological demographics are outlined in Table 1 and are stratified by the presence of sarcomatoid and/or rhabdoid differentiation. There was no significant difference in gender, age, thrombus level, histological subtype, T stage, nodal status or adjuvant target therapy between the patients with sarcomatoid and/or rhabdoid differentiation and the patients with pure RCC. RCC with sarcomatoid and/or rhabdoid differentiation tended to have a higher incidence of synchronous metastasis than pure RCC, but this difference was not significant (39.3 vs 21.6%, *p* = 0.060). However, RCC with sarcomatoid and/or rhabdoid differentiation more frequently had larger tumour size (median 8.5 vs 10.4 cm, *p* = 0.012) and higher blood transfusion (median 1600 vs 400 cc, *p* = 0.038) than pure RCC. Similarly, RCC with sarcomatoid and/or rhabdoid differentiation more frequently displayed high-grade disease (84.6 vs 59.6%, *p* = 0.018) and tumour necrosis (71.4 vs 45.4%, *p* = 0.015).

**Table 1:** Clinicopathological demographics of patients treated surgically for RCC with vena caval tumour thrombus

Characteristics	Pure RCC (*n* = 97)	RCC with sarcomatoid and/or rhabdoid differentiation (*n* = 28)	*p*
Age (years), median (IQR)	60 (53–67)	55 (52–63)	0.205
Gender, n (%)			0.614
Male	68 (70.1)	21 (75.0)	
Female	29 (29.9)	7 (25.0)	
Tumour side, n (%)			0.510
Left	25 (25.8)	9 (32.1)	
Right	72 (74.2)	19 (67.9)	
Tumour size (cm), mean ± SD	8.5 ± 3.2	10.4 ± 4.1	0.012
Thrombus level (Mayo), n (%)			0.790 0.038 0.319
I	29 (29.9)	9 (32.1)	
II	40 (41.2)	9 (32.1)	
III	19 (19.6)	6 (21.4)	
IV	9 (9.3)	4 (14.3)	
Blood transfusion (cc), median (IQR)	400 (0–2000)	1600 (400–3200)	
Tumour stage, n (%)			
T3b	84 (86.6)	21 (75.0)	
T3c	9 (9.3)	4 (14.3)	
T4	4 (4.1)	3 (10.7)	
Nodal status, n (%)			1.000
N0/Nx	88 (90.7)	26 (92.9)	
N+	9 (9.3)	2 (7.1)	
Metastatic status, n (%)			0.060
M0	76 (78.4)	17 (60.7)	
M1	21 (21.6)	11 (39.3)	
Metastatic sites, n (%)			0.701
Lung	16 (64.0)	8 (57.1)	
Bone	6 (24.0)	5 (35.7)	
Liver	3 (12.0)	1 (7.1)	
Histologic subtype, n (%)			0.454
Clear cell RCC	77 (79.4)	24 (85.7)	
Non-clear cell RCC	20 (20.6)	4 (14.3)	
Fuhrman grade, n (%) (*n* = 120)			0.018
1–2	38 (40.4)	4 (15.4)	
3–4	56 (59.6)	22 (84.6)	
Tumour necrosis, n (%)			0.015
Absent	53 (54.6)	8 (28.6)	
Present	44 (45.4)	20 (71.4)	
Adjuvant target therapy	38(39.2)	11(39.3)	0.992

*RCC* renal cell carcinoma, *IQR* interquartile range, *SD* standard deviation

### Impact of sarcomatoid and rhabdoid differentiation

**Fig. 1:**
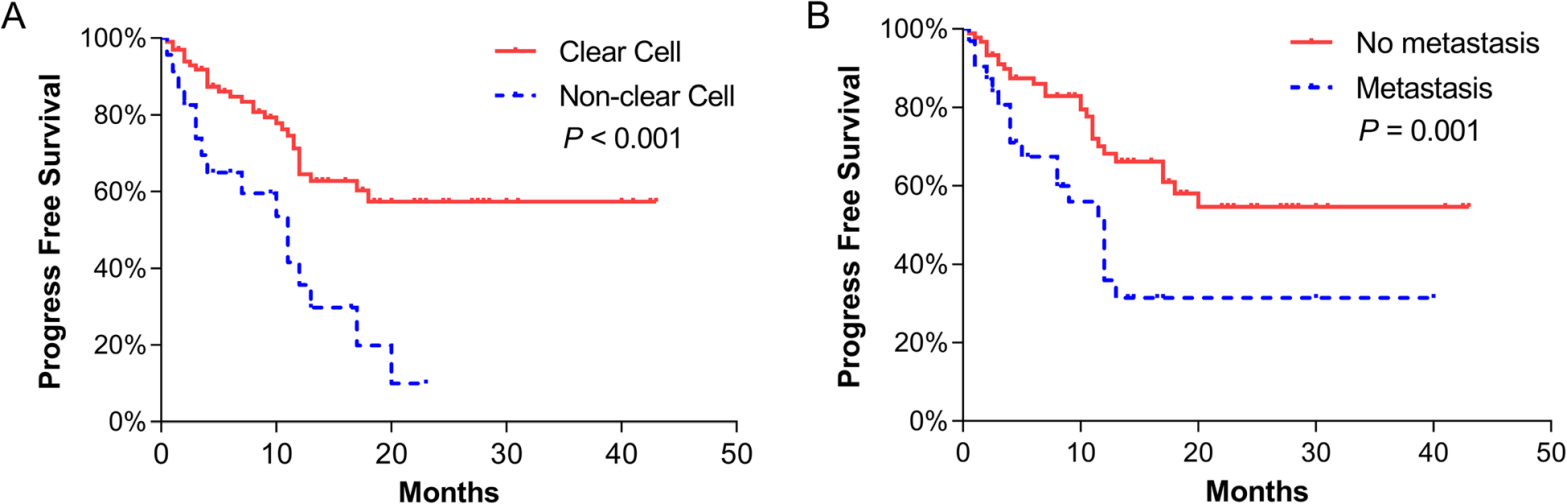
Kaplan–Meier curves of progression-free survival stratified by histologic subtype (**a**) and metastatic status (**b**)

**Fig. 2:**
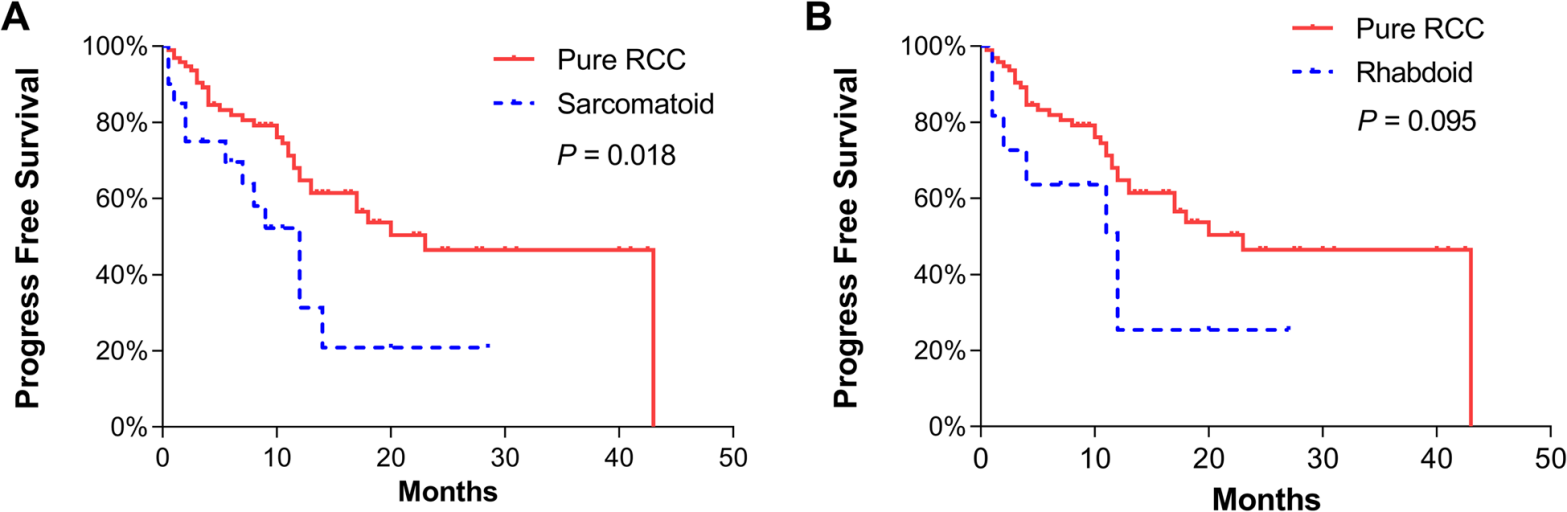
Kaplan–Meier curves of progression-free survival for patients with pure renal cell carcinoma (RCC) vs RCC with sarcomatoid differentiation (**a**) and pure RCC vs RCC with rhabdoid differentiation (**b**)

The mean follow-up was 13.6 ± 10.1 months. At the last follow-up, 55 (44.0%) patients had reported disease progression with a mean PFS of 22.7 ± 1.9 months and 22 (17.6%) had died as a consequence of RCC, with a mean CSS of 33.1 ± 1.8 months. The perioperative mortality within 90 days was 10.4% (13/125). One of them died of intraoperative pulmonary embolism. Based on the Kaplan–Meier survival analysis, non-clear cell RCC (mean 10.1 vs 26.3 months, *p* < 0.001) and metastasis (mean 14.2 vs 26.3 months, *p* = 0.001) were significantly associated with PFS (Fig. 1). Compared to pure RCC, RCC with sarcomatoid differentiation (mean 11.9 vs 25.2 months, *p* = 0.018), but not RCC with rhabdoid differentiation (mean 12.1 vs 25.2 months, *p* = 0.095), had significantly worse PFS (Fig. 2). In the univariate analysis, metastasis (HR 2.48, *p* = 0.001), non-clear cell RCC (HR 2.73, *p* = 0.001) and sarcomatoid differentiation (HR 1.99, *p* = 0.037) were significant predictors of PFS (Additional file 1: Table S1). In the multivariate analysis, metastasis (HR 2.64, *p* = 0.001), non-clear cell RCC (HR 3.23, *p* < 0.001) and sarcomatoid differentiation (HR 2.08, *p* = 0.029) all remained independent predictors of PFS (Additional file 1: Table S1). We did not include T stage in the model due to the important collinearity between T stage and thrombus level.

**Fig. 3:**
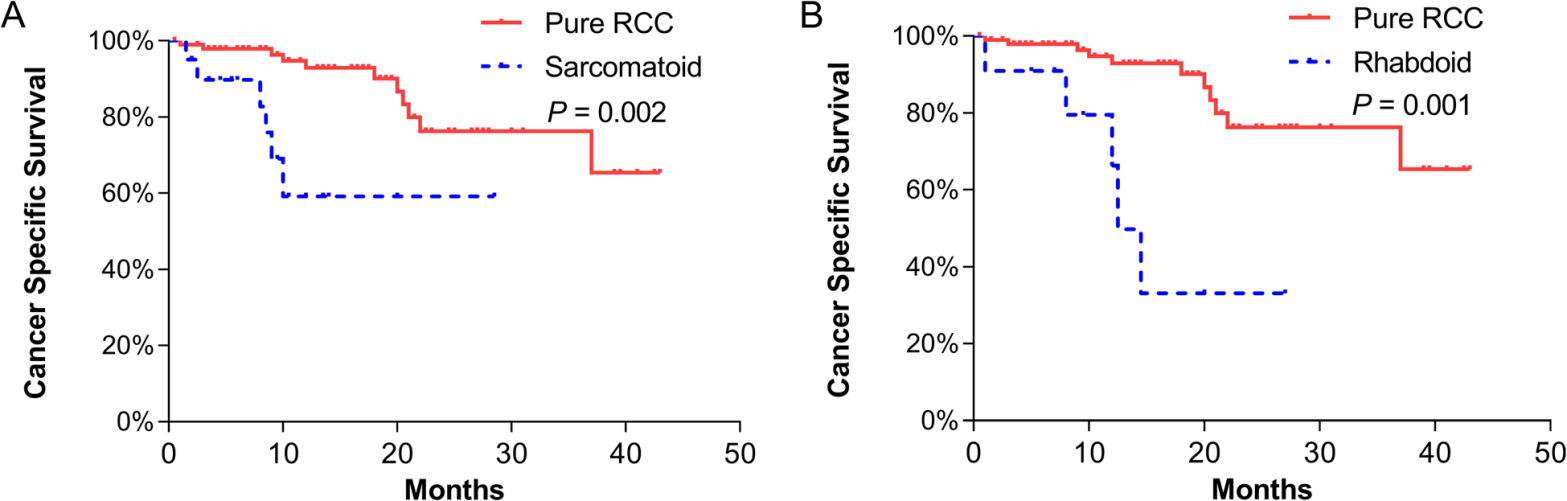
Kaplan–Meier curves of cancer-specific survival for patients with pure renal cell carcinoma (RCC) vs RCC with sarcomatoid differentiation (**a**) and pure RCC vs RCC with rhabdoid differentiation (**b**)

Based on the Kaplan–Meier survival analysis, RCC with sarcomatoid differentiation (mean 19.8 vs 35.3 months, *p* = 0.002) and rhabdoid differentiation (mean 16.0 vs 35.7 months, *p* = 0.001) had significantly worse CSS than pure RCC (Fig. 3). Metastasis was also significantly associated with CSS (mean 26.1 vs 35.9 months, *p* = 0.010). Thrombus level and histologic subtype were not associated with CSS. In the univariate analysis, sarcomatoid differentiation (HR 3.54, *p* = 0.011), rhabdoid differentiation (HR 3.82, *p* = 0.009), metastasis (HR 2.88, *p* = 0.014) and blood transfusion (HR 1.36, *p* = 0.017) were significant predictors of CSS (Table 2). In the multivariate analysis, sarcomatoid differentiation (HR 3.90, *p* = 0.008), rhabdoid differentiation (HR 3.01, *p* = 0.042), metastasis (HR 3.87, *p* = 0.004) and blood transfusion (HR 1.34, *p* = 0.041) all remained significant independent predictors of CSS (Table 2). In the univariate and multivariate analysis only blood transfusion was both significant predictor of perioperative mortality within 90 days (Additional file 1: Table S2).

**Table 2:** Univariate and multivariate Cox proportional hazard regression analyses of CSS

	Univariate		Multivariate	
	HR (95% CI)	*p*	HR (95% CI)	*p*
Gender, female	1.07 (0.43–2.66)	0.878		
Age (years)	0.99 (0.95–1.03)	0.620		
Tumour size (cm)	1.06 (0.95–1.18)	0.323		
Thrombus level				
I	Ref.			
II	1.09 (0.37–3.15)	0.881		
III	1.83 (0.61–5.46)	0.280		
IV	0.57 (0.07–4.73)	0.601		
Blood transfusion (10^3^ cc)	1.36 (1.06–1.76)	0.017	1.34 (1.01–1.77)	0.041
Lymph node involvement	1.96 (0.58–6.67)	0.279		
Metastasis	2.88 (1.24–6.70)	0.014	3.87 (1.56–9.61)	0.004
Histologic subtype				
Clear cell RCC	Ref.			
Non-clear cell RCC	1.87 (0.72–4.85)	0.196		
Sarcomatoid differentiation	3.54 (1.34–9.38)	0.011	3.90 (1.42–10.71)	0.008
Rhabdoid differentiation	3.82 (1.39–10.51)	0.009	3.01 (1.04–8.74)	0.042
Fuhrman grade				
1–2	Ref.			
3–4	1.47 (0.57–3.76)	0.423		
Tumour necrosis	1.78 (0.75–4.24)	0.191		
Adjuvant target therapy	0.55 (0.23–1.36)	0.199		

*CSS* cancer specific survival, *RCC* renal cell carcinoma

## Discussion

RCC with sarcomatoid differentiation was first reported as a distinct histologic subtype termed sarcomatoid RCC in 1968 [15]. Subsequent studies confirmed that sarcomatoid RCC can occur in all subtypes of RCC, but it does have a higher incidence in clear cell RCC [4, 6, 16]. Sarcomatoid differentiation is currently considered a rare histologic variant that predicts aggressive behaviour and poor prognosis. According to previous studies, RCC with sarcomatoid differentiation more frequently has larger tumour size, higher risk of necrosis and higher tumour stage and grade [6, 7, 16], which is consistent with the results of our study. In our cohort of RCC with vena caval tumour thrombus treated surgically, the presence of sarcomatoid differentiation in RCC was found to be an independent predictor for PFS and CSS after adjusting for other known prognostic factors. The association between sarcomatoid differentiation with poor oncologic outcomes has been consistently confirmed by many previous studies [5, 6, 16, 17]. Using the Surveillance, Epidemiology, and End Results–Medicare database, Trudeau et al. [7] identified one of the largest RCC cohorts including 234 RCCs with sarcomatoid differentiation. The results of that study showed that RCC with sarcomatoid differentiation has a worse 5-year CSS compared to pure clear cell RCC (67% vs 14%).

RCC with sarcomatoid differentiation is classified as grade 4 by the WHO/ISUP grading system [11]. However, grade 4 RCC with sarcomatoid differentiation has significantly worse CSS than grade 4 RCC without differentiation [10, 18]. We believe that the equivalence of sarcomatoid differentiation and grade 4 classification in RCC may underestimate the prognostic value of sarcomatoid differentiation. Furthermore, Adibi et al. [19] found that the percentage of sarcomatoid differentiation (PSD) was a prognostic factor for overall survival in RCC. Zhang et al. [20] suggested that PSD was an independent predictor of prognosis. However, the prognostic value of PSD in patients with RCC is still under debate [21, 22]. Our study failed to include PSD in the multivariate analysis model due to insufficient data. Insufficient pathologic material can result in incomplete and inaccurate assessment of PSD in retrospective studies. This may be one explanation for the conflicting conclusions of the above studies.

Rhabdoid differentiation, which can arise in any histologic subtype of RCC, including clear cell, papillary, chromophobe and unclassified RCC, may be a prognostic variation of RCC, similar to sarcomatoid differentiation. However, rhabdoid differentiation has not been studied as thoroughly as sarcomatoid differentiation. Gökden et al. [9] reported an incidence of rhabdoid differentiation of 4.7% and revealed associations between rhabdoid differentiation and increased grade and stage for the first time. Delahunt et al. [11] reviewed previous studies that reported survivals ranging from 8 to 31 months. In a more recent study of grade 4 RCC, 45 (28.3%) cases had rhabdoid differentiation, with a median CSS of 3.8 years [18]. To our knowledge, our study is the first to evaluate the prognostic impact of sarcomatoid differentiation and rhabdoid differentiation in RCC with vena caval tumour thrombus. We identified an incidence of rhabdoid differentiation of 8.8%, and our results supported the above hypothesis that rhabdoid differentiation in RCC is associated with adverse prognostic factors. Furthermore, we confirmed that rhabdoid differentiation in RCC is a predictor of CSS independent from sarcomatoid differentiation, thrombus level and other prognostic variables. In a prior cohort of 49 clear cell RCCs with rhabdoid differentiation, the presence of rhabdoid differentiation was shown to be an independent predictor of poor prognosis, which is consistent with the results of our study [23].

In contrast, a study of grade 4 RCC showed that rhabdoid differentiation alone was not associated with worse CSS [18]. To our knowledge, the study by Zhang et al. [10] with 111 cases and a 2-year survival of 46% is the largest reported to date; it demonstrated that RCC with rhabdoid differentiation confers an increased risk of death compared to grade 3 RCC. However, the multivariate subgroup analysis of grade 4 RCC revealed that rhabdoid differentiation was not associated with CSS. The existing studies have consistently supported the incorporation of sarcomatoid differentiation and/or rhabdoid differentiation into grade 4 RCC to improve outcome prediction. However, we suggest that it is inappropriate to treat sarcomatoid differentiation and rhabdoid differentiation equally when evaluating the prognosis of RCC.

Furthermore, we found histologic subtype to be a significant predictor of PFS and CSS, in keeping with previous studies [24, 25]. However, studies of grade 4 RCC accounting for sarcomatoid differentiation and/or rhabdoid differentiation failed to show an independent association between histologic subtype and prognosis [18, 20]. Since the cohorts only included grade 4 RCC, some non-clear cell RCCs that could not be graded using the existing Fuhrman classification system may have been excluded generating selection bias. The small sample for the sarcomatoid differentiation and rhabdoid differentiation cohorts may have been insufficient to detect differences in prognosis between the histologic subtypes. In our study, potential predictors, such as thrombus level and tumour necrosis, were not significantly associated with PFS or CSS. In contrast, most recent studies have supported the impact of thrombus level on oncologic outcomes in RCC with vena caval tumour thrombus [17, 24, 26], although some results are conflicting [27]. Currently, the prognostic significance of tumour necrosis is less certain. A study based on 3017 cases of clear cell RCC showed that the WHO/ISUP grading system achieves a better predictive ability for prognosis when the presence of tumour necrosis is incorporated [28]. However, in several studies, tumour necrosis was not shown to be an independent predictor of oncologic outcomes [18, 23, 26]. Compared to the cytokine therapy era, the targeted therapy era has achieved significant improvement in survival in RCC. However, RCC with sarcomatoid differentiation has been shown to have a poor response to targeted therapy [4, 29], and this may influence prognosis and survival.

Our study has some limitations, including its retrospective nature, single-centre experience and relatively shorter follow-up compared to previous studies. Although multivariate analyses were used to identify independent predictors of PFS and CSS, it is possible that unmeasured differences existed considering the small sample of our study. In addition, we did not perform lymph node dissection routinely for all patients which may have reduced the reliability of our results regarding the prognostic impact of lymph node involvement. Finally, there was some heterogeneity in treatment after surgery which can result in different oncologic outcomes.

## Conclusion

Our study shows that sarcomatoid differentiation and rhabdoid differentiation are associated with worse CSS in patients with RCC and vena caval tumour thrombus treated surgically. Furthermore, RCC with sarcomatoid differentiation was an independent predictor for worse PFS. Blood transfusion was an important predictor of early perioperative mortality.

## Supplementary information

**Additional file 1: Table S1.** Univariate and multivariate Cox proportional hazard regression analyses of PFS. **Table S2.** Univariate and multivariate logistic regression analyses of perioperative mortality within 90 days.

## Data Availability

The datasets used and/or analysed during the current study are available from the corresponding author on reasonable request.

## Abbreviations

CSS: Cancer-specific survival
ISUP: International Society of Urological Pathology
IVC: Inferior vena cava
PFS: Progression-free survival
PSD: Percentage of sarcomatoid differentiation
RCC: Renal cell carcinoma
WHO: World Health Organization
