# Supplementary material for: Impact of sarcomatoid differentiation and rhabdoid differentiation on prognosis for renal cell carcinoma with vena caval tumour thrombus treated surgically
Source: BMC Urol. 2020 Feb 18;20:14. doi: 10.1186/s12894-020-0584-z (PMC7029456; doi:10.1186/s12894-020-0584-z)
Supplement: Supplementary file 1 — Additional file 1: Table S1. Univariate and multivariate Cox proportional hazard regression analyses of PFS. Table S2. Univariate and multivariate logistic regression analyses of perioperative mortality within 90 days. [file 12894_2020_584_MOESM1_ESM.docx]

| **Supplement Table 1** Univariate and multivariate Cox proportional hazard regression analyses of PFS | | | | | |
| --- | --- | --- | --- | --- | --- |
|  | Univariate | |  | Multivariate | |
|  | HR (95% CI) | *p* |  | HR (95% CI) | *p* |
| Gender, female  Age (years)  Tumour size (cm)  Thrombus level  I  II  III  IV  Blood transfusion (10^3^ cc)  Lymph node involvement  Metastasis  Histologic subtype  Clear cell RCC  Non-clear cell RCC  Sarcomatoid differentiation  Rhabdoid differentiation  Fuhrman grade  1-2  3-4  Tumour necrosis  Adjuvant target therapy | 1.20 (0.68-2.11)  0.99 (0.96-1.01)  0.98 (0.91-1.06)  Ref.  1.16 (0.61-2.21)  1.43 (0.69-2.95)  0.67 (0.20-2.29)  1.13 (0.95-1.34)  2.01 (0.91-4.44)  2.48 (1.44-4.27)  Ref.  2.73 (1.54-4.82)  1.99 (1.04-3.79)  1.68 (0.76-3.72)  Ref.  1.61 (0.86-3.03)  1.71 (0.99-2.96)  0.95 (0.55-1.64) | 0.201  0.213  0.610  0.643  0.333  0.525  0.156  0.086  **0.001**  **0.001**  **0.037**  0.200  0.137  0.057  0.858 |  | 2.64 (1.81-5.78)  Ref.  3.23 (1.81-5.78)  2.08 (1.08-4.01) | **0.001**  **<0.001**  **0.029** |
| PFS progression-free survival, RCC renal cell carcinoma | | | | | |

| **Supplement Table 2** Univariate and multivariate logistic regression analyses of perioperative mortality within 90 days | | | | | |
| --- | --- | --- | --- | --- | --- |
|  | Univariate | |  | Multivariate | |
|  | OR (95% CI) | *p* |  | OR (95% CI) | *p* |
| Gender, female  Age (years)  Tumour size (cm)  Thrombus level  I  II  III  IV  Blood transfusion (10^3^ cc)  Lymph node involvement  Metastasis  Histologic subtype  Clear cell RCC  Non-clear cell RCC  Sarcomatoid differentiation  Rhabdoid differentiation  Fuhrman grade  1-2  3-4  Tumour necrosis  Adjuvant target therapy | 4.17 (0.09-1.98)  1.00 (0.95-1.05)  1.05 (0.90-1.23)  Ref.  -  0.77 (0.14-4.34)  1.38 (0.23-8.30)  1.85 (1.29-2.66)  2.08 (0.40-10.87)  1.33 (0.38-4.67)  Ref.  0.74 (0.15-3.60)  1.68 (0.42-6.73)  0.85 (0.10-7.23)  Ref.  2.94 (0.61-14.10)  0.80 (0.25-2.53)  0.11 (0.01-0.88) | 0.272  0.916  0.547  1.000  0.765  0.728  **0.001**  0.385  0.653  0.713  0.466  0.882  0.177  0.701  **0.038** |  | 1.89 (1.28-2.79)  0.12 (0.01-1.00) | **0.001**  0.050 |
| RCC renal cell carcinoma | | | | | |
